# Enhancing the Energy Absorption Performance of 3D-Printed CF/TPU Composite Materials by Introducing a “Rigid–Elastic” Structure Through Multi-Scale Synergies

**DOI:** 10.3390/polym17131880

**Published:** 2025-07-06

**Authors:** Xuanyu Zhou, He Ouyang, Yuan Zhang, Ziqiang Zhu, Zhen Wang, Zirui Cheng, Yubing Hu, Yanan Zhang

**Affiliations:** 1 Jiangsu Collaborative Innovation Center for Advanced Inorganic Function Composites, Nanjing Tech University, Nanjing 211816, China; 202361203140@njtech.edu.cn; 2 National Special Superfine Powder Engineering Research Center of China, Nanjing University of Science and Technology, Nanjing 210014, China; ouyanghe@njust.edu.cn (H.O.); 123103010315@njust.edu.cn (Y.Z.); wangzhen2012@njust.edu.cn (Z.W.); chengzr@njust.edu.cn (Z.C.)

**Keywords:** 3D printing, rigid–elastic structure, multi-scale synergies, mechanical properties, energy absorption

## Abstract

Thermoplastic polyurethane (TPU) combines elastomeric and thermoplastic properties but suffers from insufficient rigidity and strength for structural applications. Herein, we developed novel carbon fiber-reinforced TPU (CF/TPU) composites filaments and utilize melt extrusion for 3D printing to maintain elasticity, while achieving enhanced stiffness and strength through multi scale-the control of fiber content and optimization of printing parameters, reaching a rigid–elastic balance. A systematic evaluation of CF content (0–25%) and printing parameters revealed optimal performance to be at 220–230 °C and 40 mm/s for ensuring proper flow to wet fibers without polymer degradation. Compared with TPU, 20% CF/TPU exhibited 63.65%, 105.51%, and 93.69% improvements in tensile, compressive, and impact strength, respectively, alongside 70.88% and 72.92% enhancements in compression and impact energy absorption. This work establishes a fundamental framework for developing rigid–elastic hybrid materials with tailored energy absorption capabilities through rational material design and optimized additive manufacturing processes.

## 1. Introduction

Modern battlefield threats demand helmet liners that simultaneously mitigate blunt impact trauma (e.g., ballistic backface deformation) and attenuate chronic blast-induced traumatic brain injury (bTBI) [1], while maintaining ultralight mass (<600 g). Conventional expanded polystyrene (EPS) foams exhibit brittle collapse under multi-hit loading [2], and non-Newtonian shear-thickening fluids suffer from temperature-dependent rheological instability [3]. CF/TPU composites with architectured auxetic geometries emerge as a paradigm-shifting solution, leveraging strain-rate-sensitive TPU matrix dynamics and stochastic fiber network energy dissipation [4].

Thermoplastic polyurethane (TPU) is distinguished by exceptional elasticity (>800% elongation at break), shape recovery (>90% after 50% strain), energy absorption rivaling traditional elastomers, and superior damping efficiency (tanδ ≈ 0.25 at 1 Hz), outperforming silicone rubber in vibration isolation [5]. Despite these advantages over conventional FDM polymers like PLA and ABS, research on 3D-printed TPU remains sparse (<10% of polymer AM studies), hindering broader engineering adoption. [6,7,8,9,10,11] However, structural applications are limited by inherent tensile strength (<50 MPa) and compressive strength (<30 MPa at 25% strain), compounded by complex rheology (melt viscosity >1000 Pa·s at 100 s^−1^), causing extrusion inconsistencies like nozzle clogging. Carbon fiber (CF) reinforcement, particularly with PAN-based short fibers (50–200 μm), emerges as a transformative solution, leveraging CF’s high axial thermal conductivity (400 W/m·K) and oxidative stability (550 °C) [12,13]. This induces a unique rigid–elastic duality; Yuhua Zhao demonstrated tensile strength increase while maintaining elongation. Critically, short CF offers significant processing advantages over continuous fiber systems: 40–60% reduced fabrication time by eliminating alignment steps [14], isotropic mechanical properties (<15% XY-plane strength variation), and an unexpected 30–50% melt viscosity reduction via fiber-induced shear thinning, directly mitigating extrusion challenges. These synergistic enhancements—retained elasticity with boosted stiffness and streamlined processability—are driving rapid adoption in demanding sectors like automotive energy-absorbing components and next-generation athletic footwear, where impact resistance and weight savings are paramount [15,16,17,18].

Additive manufacturing (AM) offers unprecedented design freedom for fabricating complex geometries inherent to emerging TPU applications, from topology-optimized damping structures to intricate soft robotic actuators [19]. However, the transition of high-performance carbon fiber-reinforced thermoplastic polyurethane (CF/TPU) composites into reliable AM processes faces significant scientific hurdles [20]. While short CF reinforcement synergistically enhances TPU mechanical properties, its integration into fused filament fabrication (FFF) introduces multifaceted interdependencies. The printing process itself becomes a critical determinant of final performance; CF orientation, dispersion, and interfacial adhesion are acutely sensitive to thermal gradients and shear histories during extrusion and deposition. Key parameters—including nozzle temperature (dictating polymer flow and degradation), print speed (influencing crystallization kinetics and fiber alignment), layer adhesion (governed by thermal penetration), and volumetric flow rate (modulating shear thinning efficacy)—collectively orchestrate microstructure development. Presently, the quantitative relationships between these parameters and the resultant thermomechanical properties of CF/TPU AM components remain incompletely characterized, limiting predictability and reproducibility. Systematic investigation of the processing–structure–property nexus is therefore imperative to unlock the full potential of these advanced elastomeric composites in demanding functional applications [19,21,22,23,24].

Herein, the critical role of material composition in determining manufactured components mechanical properties has been largely overlooked. As demonstrated in this study, our primary objectives cover three steps: to develop CF/TPU composite filaments integrating both elastic and rigid characteristics; to systematically investigate how carbon fiber mass fraction governs microstructure, thermal behavior, and printability; and to study the dynamic and static mechanical properties of materials and analyze the rigid elastic synergy effect. Multi-scale mechanical characterization was carried out through tensile testing, cyclic compression analysis, and split Hopkinson pressure bar (SHPB) impact evaluation. This comprehensive approach establishes a new paradigm for designing rigid elastic hybrid materials with customized energy absorption capabilities.

## 2. Experimental

### 2.1. Materials

Thermoplastic polyurethane TPU (Germany Elastollan, 95A injection molding particles) and the CF (T300, 3 K, diameter about 7 μm) were received from Siweiqi Carbon Co., Ltd., Wuxi, China.

### 2.2. Preparation of CF/TPU Composite

**Figure 1:**
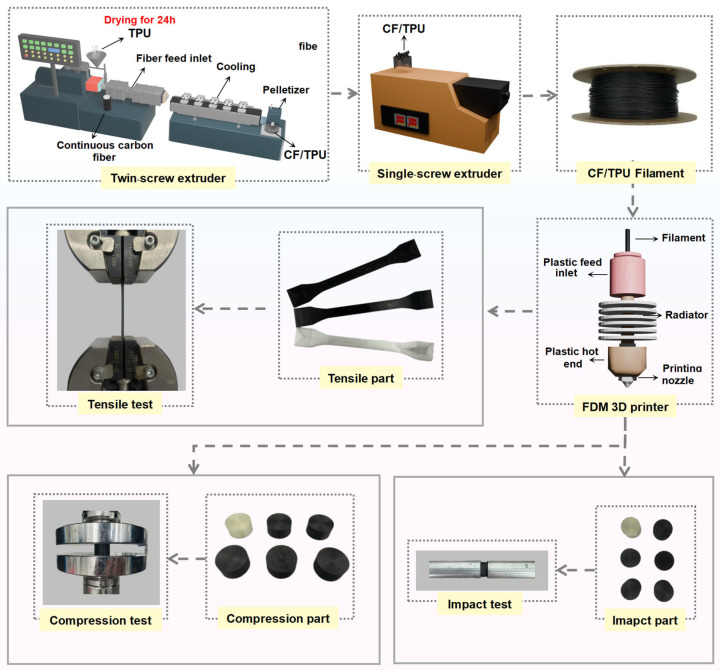
Process for composite filament preparation and 3D printing sample testing.

This study employed a sequential methodology (Figure 1). The first priority is the manufacturing process of CF/TPU composite filaments. All production was conducted at China National Powder Research Center under subtropical monsoon conditions (average relative humidity > 75%), necessitating rigorous moisture control. Matrix materials and TPU pellets were pre-dried in a forced-air convection oven (DHG-9426A, Shanghai Jinghong Co., Ltd. Shanghai, China) at 75 °C for 24 h to achieve <0.1% moisture content. Granulation was performed using a co-rotating twin-screw extruder (SHJ-20, China Nanjing Juli Chemical Machinery Co., Ltd. Nanjing, China) featuring seven precisely controlled thermal zones, including six barrel zones set to175, 205, 205, 205, 195, and 195 °C. The dead zone was set to 210 °C. This thermal profile was optimized to ensure complete polymer melting while preventing feed-throat blockage, with Zone 1 maintained at lower temperature to avoid premature melt adhesion. Carbon fibers were introduced at Zone 3 after TPU matrix plastication, enabling homogeneous dispersion. By adjusting the feed rate and screw speed [25], TPU was uniformly mixed with neat CF at different mass fractions (0, 5, 10, 15, 20, and 25%). Specific parameters are presented in Table 1. The extruded CF/TPU composite filaments were pelletized into particle with a size of 2–3 mm using a pelletizer [26]. The process of the preparation of CF/TPU particles is provided in the Supplementary Materials, Figure S1.

After pelletizing, the CF/TPU composite masterbatch was placed in an oven at 50 °C for drying for 6 h. The composite pellets were subsequently melt-extruded into 1.75 mm diameter filaments using a Wellzoom single-screw desktop extruder (Figure 1) with two zone-specific temperature profiles (Table 2). Precise thermal management (±1 °C) in the die and heating zones yielded filaments with diameter variation ≤±2.9% (1.75 ± 0.05 mm), This dimensional consistency meets commercial FDM printer specifications, with surface roughness (Ra) < 5 μm [19,27] across all filaments.

### 2.3. 3D Printing of Composite Samples

**Figure 2:**
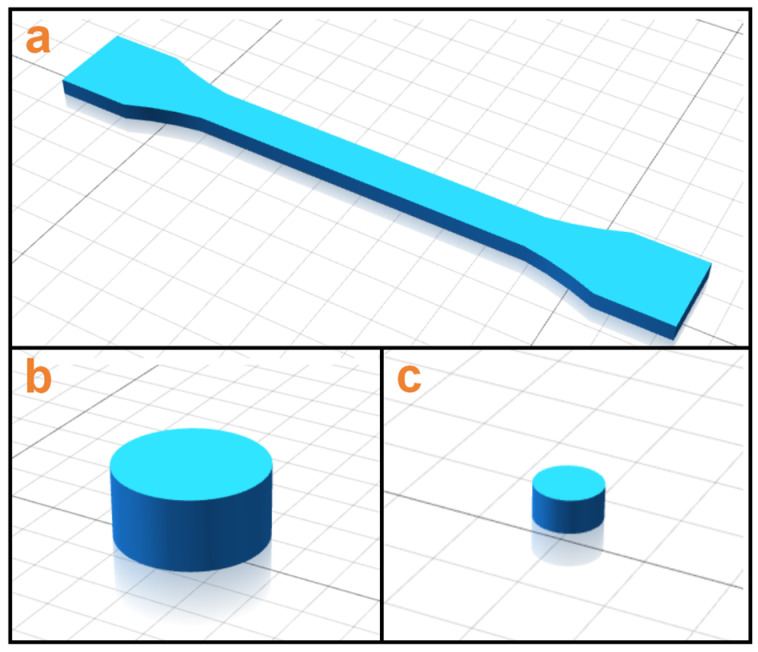
3D models of specimens used in this study: (**a**) tensile specimens, (**b**) compression specimens, (**c**) impact specimens.

The required specimens (Figure 1) for various characterization methods used in this study were printed using Anisoprint 3D printer (Composer A4, Anisoprint Inc., Esch-sur-Alzette, Luxembourg) equipped with a heated build platform and chamber. The fabricated filaments were fed into the extruder of the AM machine attached to a hardened steel nozzle with a diameter of 0.4 mm. The heat bed leveling initiates by preheating the build plate to 60 °C, followed by thorough cleaning with isopropyl alcohol and a microfiber cloth. The automated AutoLevel routine then deploys a strain-gauge probe to map 25 grid points, detecting Z-deviations within ±5 µm. Manually, bed screws use real-time servo feedback to achieve uniform contact pressure (0.15 ± 0.02 N). Critical dual-Z calibration is conducted as follows; the thermoplastic nozzle height is set using a 0.1 mm certified shim (slight drag test), while the fiber module is offset +0.05 mm above it. A validation test print confirms first-layer uniformity (0.20 ± 0.01 mm via micrometer), with fiber feed disabled throughout to prevent carbon debris contamination. The 3D model of the STL format specimen (as shown in Figure 2) is sliced using Aura 3D 2.2.3 software to achieve 3D manufacturing. The component filling rate is 100% and layer height is 0.4 mm.

### 2.4. Thermal Characterization of CF/TPU Composite Filaments

#### 2.4.1. Analysis Using a Differential Scanning Calorimetry (DSC)

The thermal characteristics of CF/TPU composites and matrix materials (5.0–5.2 mg) were analyzed with a DSC instrument (Mettler Toledo, TGA/DSC 3+). The sample was initially heated to 270 °C at a rate of 10 °C/min and maintained at 270 °C for 5 min to remove any previous thermal effects. The crystallization temperature (Tc), crystallization enthalpy (ΔHc) and melting temperature (Tm) can be calculated from the peak values of the curve.

#### 2.4.2. Determination of Melt Flow Rate

According to the melting extrusion principle of FDM 3D printer, different temperatures may cause inconsistent discharge of the melt when sending the filament to the printer for extrusion, resulting in inconsistent product dimensions, which will lead to changes in product specifications. To investigate the effect of carbon fiber content on the flowability of TPU and determine the printing temperature range, a solution flow rate meter (TM2101-T5, Shenzhen Wance Testing Machine Co., Ltd. Shenzhen, China) was used to test the melt flow index (MFI) of CF/TPU composite materials with ISO 1133-1:2022. The MFI values of commercial TPU filaments were compared to determine the printing temperature range of CF/TPU. The load used in MFI testing remains constant for all materials (2.15 kg).

### 2.5. Tests for Mechanical Properties

#### 2.5.1. 3D Printing Parameters and Tensle Tests

To systematically evaluate the processing–structure–property relationships, we investigated the effects of printing parameters and fiber content on the mechanical performance of CF/TPU composites. For all CF/TPU samples, a full factorial experimental design was implemented, testing four nozzle temperatures (220, 230, 240, and 250 °C) and three printing speeds (10, 40, and 70 mm/s). The mechanical response was characterized using dumbbell specimens (n = 5; total length = 150 mm; gauge length = 50 mm; width = 10 mm) tested at 10 mm/min on a universal testing machine (CTM100GD MTS Systems China Co., Ltd., Shanghai, China) per GB/T 1040.1-2018. Herein, six CF (0–25%) were evaluated to determine composition-property correlations, with tensile strength, modulus, and elongation at break quantified for each formulation. Fracture surface morphology was analyzed by scanning electron microscopy (SEM) (gold was sprayed on the surface of the sample in a vacuum environment) before the experiment to elucidate fiber–matrix interfacial interactions and failure mechanisms, with particular attention to fiber pull-out, matrix deformation, and void formation characteristics.

#### 2.5.2. Compression Tests

To explore the mechanical performance of CF/TPU composites under cyclic loading, we conducted compressive tests in accordance with ISO 7743 using a universal testing machine equipped with a 5000 N load cell. Cylindrical specimens (n = 5; diameter = 29 ± 0.5 mm, height = 12.5 ± 0.5 mm) with varying carbon fiber content (0–25%) were subjected to four consecutive compression cycles at 10 mm/min, each comprising loading to 25% strain followed by complete strain release. This protocol enabled quantitative characterization of key parameters including compressive strength (first-cycle peak stress), modulus, hysteresis energy (area between loading-unloading curves), energy loss (the energy difference between the first-cycle and last-cycle), and energy absorption efficiency (first-cycle energy), with particular attention to the Mullins effect evolution across cycles.

#### 2.5.3. Dynamic Impact Tests

The dynamic mechanical response of CF/TPU composites under high-strain-rate loading was investigated using a split Hopkinson pressure bar (SHPB) (Nanjing Tangshan Mechanics Experiment Equipment Co., Ltd. Nanjing, China) system with 10 mm diameter aluminum bars (E = 71 GPa, ρ = 2.8 g/cm^3^, wave speed ≈ 4970 m/s). Cylindrical specimens (8 mm diameter × 4 mm thickness) with varying carbon fiber content were tested in a setup comprising a gas gun launcher, 300 mm striker bar, 1000 mm incident/transmitter bars, and 600 mm absorber bar. Pulse shaping with copper paper (8 × 8 × 0.4 mm) minimized dispersion effects while ensuring stress equilibrium. Strain signals were acquired using metallic (incident wave) and semiconductor (transmitted wave) strain gauges coupled with a high-speed data acquisition system, enabling precise measurement of dynamic yield stress, impact strength, and energy absorption characteristics at strain rates >10^3^ s^−1^.

## 3. Results and Discussion

### 3.1. Thermal Characteristics

**Figure 3:**
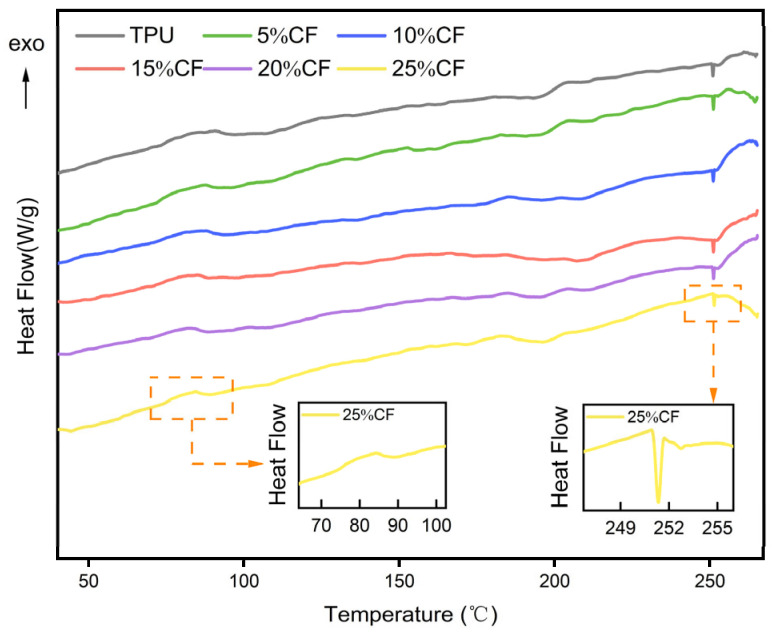
DSC curves of composites with different CF contents.

To investigate the effect of CF% on the crystallization and melting behavior of TPU, DSC was used to analyze the melt crystallization behavior of CF/TPU composites. Figure 3 shows the DSC curves of filaments with different CF contents in CF/TPU composites. Table 1 presents the key parameters, while Table 3 lists the thermal parameters *Tc*, *Tm*, ΔH_0_, Δ*Hc,* and *Xc*. The crystallinity of the TPU phase was calculated using the following formula [26,28]:

(1) $$\;Xc=\frac{\Delta Hc}{(1-\omega_{f})\Delta H_{0}}$$

In the formula, Δ*Hc* is the hard end crystallization enthalpy (J/g) of the sample, *ωf* is the mass fraction of CF, and ΔH_0_ is the theoretical melting enthalpy of 100% crystalline TPU (150.6 J/g) [29].

In Figure 3, the two local magnified images show the crystallization peak and melting peak of the TPU hard segment. It is evident that the degree of crystallinity gradually decreases. This is because carbon fibers, acting as rigid fillers, hinder the orderly arrangement of the hard segment molecular chains, thereby limiting the formation of nuclei and crystal growth, leading to a decrease in crystallinity, while the influence of small crystallinity on the mechanical properties of materials can be ignored. Additionally, the figure shows that as the CF content increases, the TC decreases. This is due to the dual effects of CF on molecular chain movement. The first is physical obstruction—CF, acting as a rigid reinforcing phase, directly hinders the sliding and orderly arrangement of molecular chains. The second is the interface adsorption effect—CF surfaces form an adsorption layer with the NCO groups of TPU through hydrogen bonding, significantly reducing the activity of the chain segments, requiring a lower temperature to overcome the energy barrier and complete nucleation. However, the addition of CF has little impact on the melting temperature of TPU.

**Figure 4:**
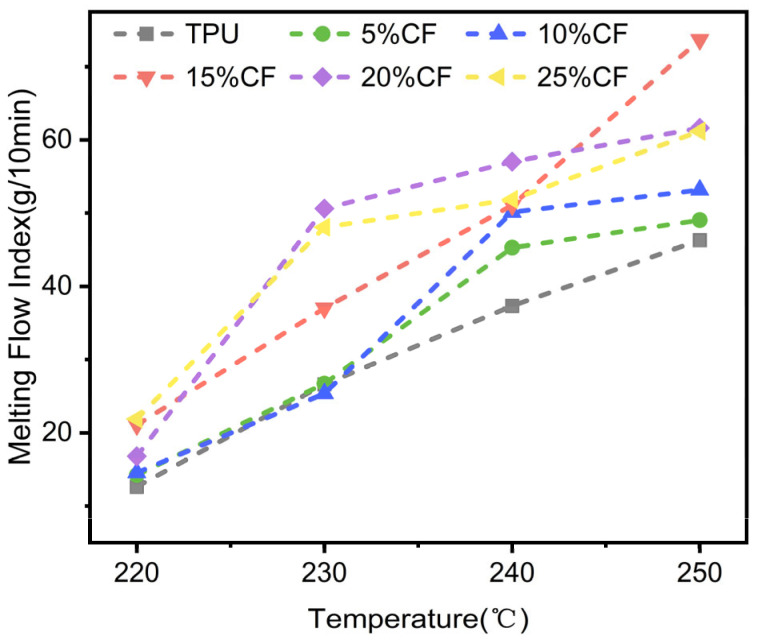
Melting flow index of CF/TPU with different fiber contents (temperature from 220 °C to 250 °C).

The CF/TPU composite material exhibits a melting temperature of 250 °C as determined from Figure 3, establishing this threshold as the maximum permissible processing temperature for 3D printing applications. Commercial Ultrafuse TPU 95A (TPU-Ultimaker, FlexiSmart, PolyFlex_95A, Filaflex_95A) demonstrates a melt flow rate (MFR) of 7–9 g/10 min under standard testing conditions (190 °C, 2.16 kg load), with recommended extrusion temperatures ranging from 210 °C to 230 °C. In this study, the printability and thermal behavior of CF/TPU composites were systematically evaluated across a temperature gradient (220, 230, 240, and 250 °C), with the resultant mechanical and morphological characteristics presented in Figure 4. Notably, the upper temperature boundary (250 °C) corresponds to the material’s thermal degradation limit, beyond which structural integrity and print fidelity are compromised.

Experimental results confirm that CF/TPU composites exhibit increased melt flow index (MFI) with rising temperature, driven by carbon fibers promoting molecular chain slippage within the TPU matrix—a key mechanism enhancing thermoplastic flow. The reinforcement facilitates interchain sliding under heating, improving melt fluidity. However, at 240 °C, excessive fiber loading (25%CF) induces agglomeration, impeding flow dynamics and reducing MFI by 18% compared to 20% CF/TPU (Figure 4). Critically, below 220 °C, the material approaches a quasi-static state in the MFI rheometer (<1 g/10 min), indicating insufficient fluidity for extrusion. Optimal printability occurs between 220–240 °C, where CF/TPU MFI aligns with commercial TPU benchmarks (5–40 g/10 min, 190–240 °C/2.16 kg)—a range empirically validated for extrusion-based additive manufacturing [19,27,30,31]. This window balances enhanced chain mobility with suppressed fiber-induced shear resistance, ensuring rheological compatibility for robust 3D printing.

### 3.2. Mechanical Properties of 3D-Printed Parts

**Figure 5:**
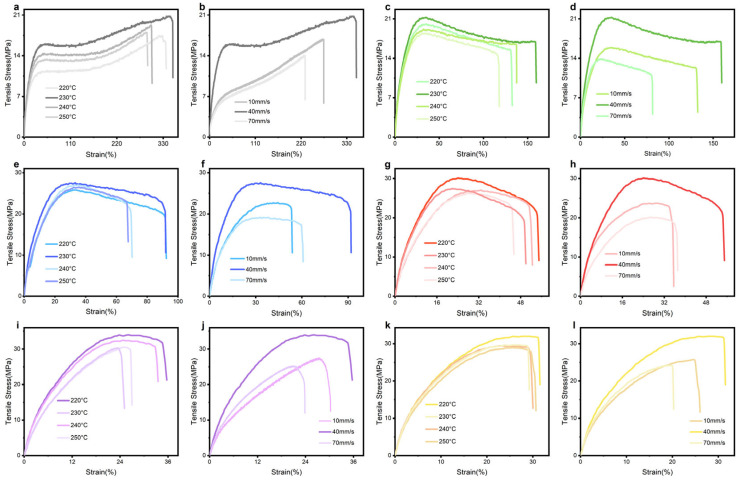
Tensile stress–strain curves of CF/TPU with different fiber contents at different printing temperatures and printing speeds: (**a**,**b**) 0% CF, (**c**,**d**) 5% CF, (**e**,**f**) 10% CF, (**g**,**h**) 15% CF, (**i**,**j**) 20% CF, (**k**,**l**) 25% CF.

To determine the optimal 3D printing parameters CF/TPU composites, tensile specimens with (0–25)% CF content were fabricated via fused deposition modeling (FDM) at a fixed print speed of 40 mm/s across nozzle temperatures spanning 220–250 °C. Tensile tests were performed on all printing specimens at different printing temperatures and speeds (Figure 5). Take Figure 4 as an example, compared with 220, 240, and 250 °C, specimens printed at 230 °C (27.60 Mpa 92.01%) exhibited 7.10%,1.96%, and 4.31% improvements in tensile stress, alongside 1.23%, 32.32%, and 37.35% enhancements in elongation at break. Maximum performance was attributed to temperature-dependent rheological behavior, elucidated by prior melt flow index (MFI) analyses. Sub-220 °C (<1 g/10 min) conditions induce premature melt solidification, elevating viscosity to levels that hinder uniform filament deposition and interfacial fusion, resulting in stress-concentrating microvoids from incomplete polymer chain entanglement. Conversely, temperatures exceeding 230 °C destabilize melt flow dynamics through excessive fluidity and shear-thinning effects, as evidenced by MFI surges at 250 °C, which disrupt dimensional accuracy via insufficient cooling time for structural stabilization [32,33]. The 220–230 °C optimum balances melt viscosity for effective fiber–matrix stress transfer and interlayer diffusion kinetics, while mitigating thermal degradation pathways, enabling robust interfacial bonding through thermally activated chain reptation without compromising melt stability.

The printing speed critically governs the thermal history and interlayer bonding dynamics of materials during fused deposition modeling (FDM). As shown in Figure 5f, tensile performance tests at 10 mm/min were conducted at three speeds (10, 40, and 70 mm/s) using the recommended fixed nozzle temperature of 230 °C from Figure 5e. The results indicated that the mechanical response was optimal at 40 mm/s. Compared to 10 mm/s and 70 mm/s, this speed increased the tensile strength by 21.53% and 43.90%, and the elongation at break by 71.09% and 51.37%, respectively. This phenomenon arises from the interplay of thermally activated molecular diffusion and viscoelastic relaxation kinetics. At 10 mm/s, prolonged thermal exposure induces excessive polymer chain degradation and localized overcooling, disrupting crystallinity and interfacial entanglement. Conversely, at 70 mm/s, insufficient melt residence time limits interlayer diffusion, while rapid cooling suppresses molecular chain alignment and fiber–matrix stress transfer in composites. The 40 mm/s regime balances these extremes, enabling adequate interdiffusion of polymer chains across deposited layers and optimal shear-induced alignment of reinforcing phases without compromising thermal stability [34,35]. Figure 5 demonstrates that the printing speed of CF/TPU with different fiber contents has a greater impact on the tensile properties of the material compared to printing temperature. The optimal printing parameters based on the comprehensive experimental results are shown in Table 4.

**Figure 6:**
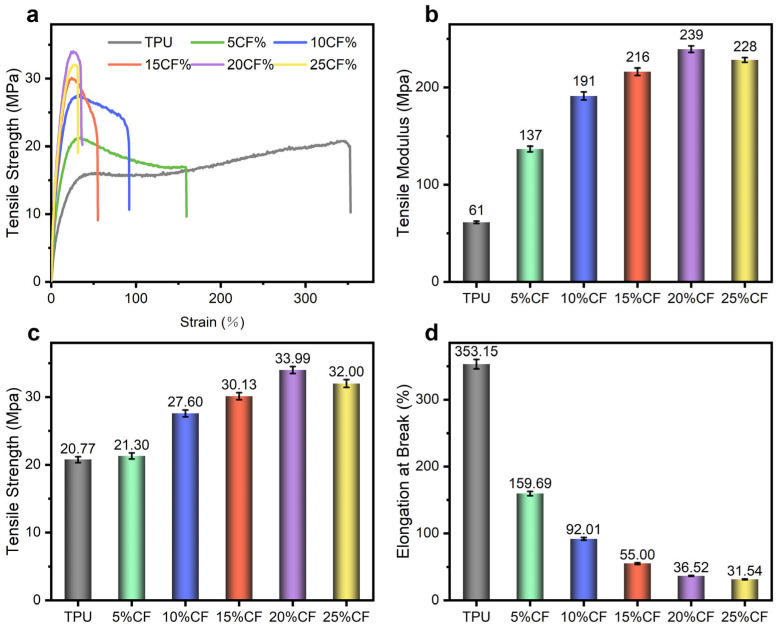
Result of tensile mechanics: (**a**) tensile stress–strain curve, (**b**) tensile modulus, (**c**) tensile strength, (**d**) elongation at break.

Based on the analysis of the optimal printing parameters mentioned above, the mechanical performance of CF/TPU composites printed at 220–230 °C/40 mm/s reveals a non-monotonic dependence on carbon fiber content (0–25%), with 20% CF delivering a peak tensile strength (Figure 6c) of 33.39 MPa (a 63.65% increase over neat TPU) and a modulus of 239 MPa (273.77% higher than TPU). This reinforcement threshold emerges from competing mechanisms. Below 20%, increasing CF content enhances stiffness through load-bearing fiber networks, elevating the modulus progressively (Figure 6b). However, elongation at break declines nonlinearly (Figure 6d)—most precipitously beyond 15—as expanding CF free volume restricts TPU chain mobility and disrupts stress transfer, culminating in 25% composites exhibiting the lowest ductility (31.54% elongation). Critically, in 25% composites, fiber agglomeration and entanglement occurs, evidenced by diminished strength and modulus, versus 20%, which offsets reinforcement gains through stress concentration sites. The 10–15% formulations achieve optimal rigid–elastic synergy, retaining 92.01% and 55.00% elongation while attaining 27.60 MPa and 30.13 MPa strength, merely 6.39 MPa and 3.86 MPa below peak values, thus balancing interfacial integrity (enabled by homogeneous dispersion) with effective stiffening [14,36].

**Figure 7:**
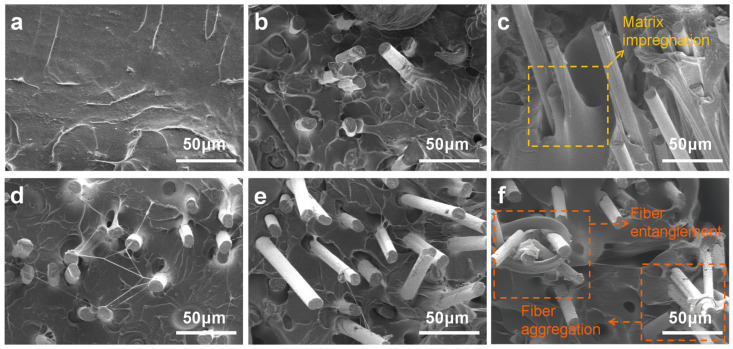
SEM images of the composite with different CF contents: (**a**) 0% CF, (**b**) 5% CF, (**c**) 10% CF, (**d**) 15% CF, (**e**) 20% CF, (**f**) 25% CF.

In order to further evaluate the reinforcement threshold emerges of CF/TPU, the cross-sectional morphologies of the composite filaments with different CF contents (0–25%) were investigated by SEM, as shown in Figure 7a–f. The neat TPU (0%CF, Figure 7a) exhibits characteristic ductile fracture morphology, featuring uniformly distributed dimples formed by extensive localized plastic deformation of the matrix, indicative of high ductility. Energy dissipation primarily occurs through molecular chain slippage and hydrogen bond rupture. At lower CF contents (5%, 10%, 15%; Figure 7b–d), short carbon fibers are well-dispersed without significant agglomeration. The TPU matrix remains the primary load-bearing constituent. Fracture surfaces show predominantly matrix plastic deformation; most fiber ends are encapsulated within the matrix, with minimal fiber pull-out, evidencing excellent fiber–matrix impregnation and wetting (Figure 7c). TPU fully coats the fibers, indicating thorough melt penetration into fiber–matrix interstices. The failure mechanism transitions to a synergistic combination of fiber fracture and matrix plastic deformation, signifying strong interfacial shear strength (IFSS). Conversely, at higher CF concentrations (20%, 25%; Figure 7e,f), the increased fiber density shifts the primary load-bearing role to the carbon fibers. The fracture mechanism is dominated by interfacial failure, manifesting as pronounced fiber pull-out with increased pull-out hole depth, resulting in brittle fracture characteristics for the composite. Moreover, Figure 7f shows the occurrence of 25% CF/TPU local fiber entanglement and agglomeration [31,37], verifying the reason for the decrease in tensile performance at 25%.

**Figure 8:**
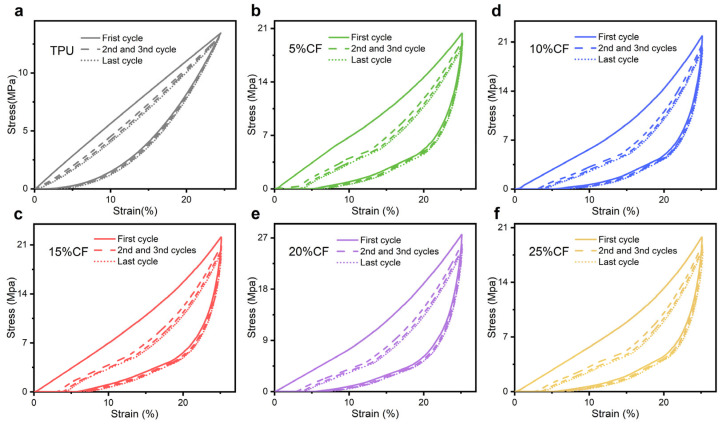
Result of compression mechanics. (**a**) 0% CF, (**b**) 5% CF, (**c**) 10% CF, (**d**) 15% CF, (**e**) 20% CF, (**f**) 25% CF.

To further investigate the influence of fiber content on rigid elastic structures, cyclic compression specimen (printing rate: 40 mm/s; nozzle temperature: 220–230 °C) tests subjected to four consecutive compression cycles at 10 mm/min, each comprising loading to 25% [18,38], elucidate the mechanical response of CF/TPU composites across varying fiber contents (Figure 8). The 20% CF composite achieves the maximum compressive strength of 27.58 MPa, demonstrating superior load-bearing capacity.

**Figure 9:**
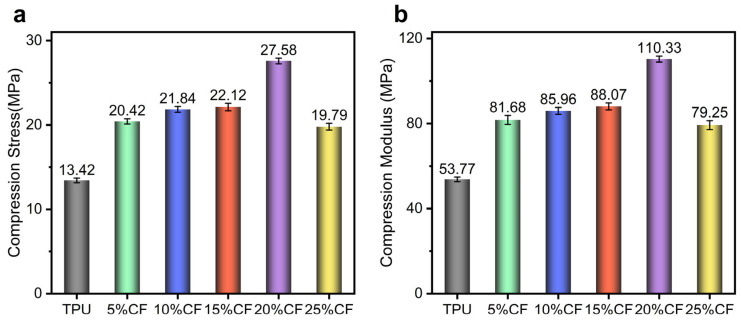
Result of compression mechanics: (**a**) compression stress; (**b**) compression modulus.

CF content has a significant reinforcement effect on the quasi-static compressive properties of TPU composites, as shown in Figure 9. Both compressive strength and modulus exhibit a monotonic enhancement with increasing CF content up to 20%, where optimal stress transfer is achieved. The 20% CF composite delivers peak performance, attaining a compressive strength of 27.58 MPa (a 105.51% increase over neat TPU) and a modulus of 110.33 MPa (135.1% higher than neat TPU). This synergistic enhancement arises from effective percolated fiber network formation and robust fiber–matrix interfacial adhesion. Beyond this critical threshold (25% CF), strength declines to 19.79 MPa and modulus drops to 85.96 MPa, indicating compromised properties due to fiber agglomeration, stress concentration sites, and reduced matrix continuity. The inverse trends in strength and modulus at 25% CF confirm the transition from reinforcement-dominated to defect-dominated behavior, highlighting the 20% formulation as the optimal balance for simultaneous stiffness and strength augmentation under compression.

**Figure 10:**
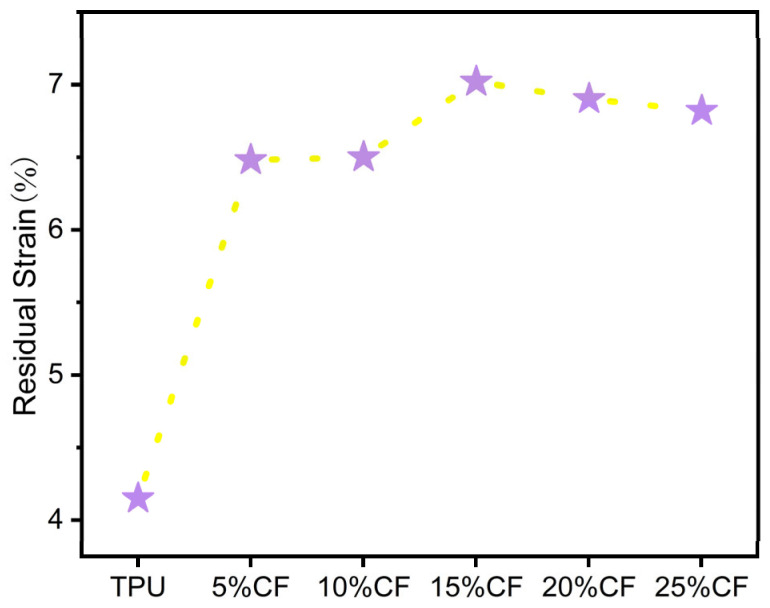
Residual strain of CF/TPU composites after cyclic compression.

The residual (Figure 10) strain (quantifying permanent deformation after unloading) exhibits a pronounced minimum at neat TPU [39], significantly lower than all other compositions. This ultra-low value signifies near-perfect elastic recovery. The slight increase in residual strain with fiber incorporation stems from the evolving damage dynamics within the polymer network during compression. While the TPU matrix undergoes repeated elastic deformation and chain fracture events, the introduction of carbon fibers disrupts the continuity of the molecular chain network. This disruption amplifies localized stress concentrations, promoting additional chain breakage. Consequently, the accumulation of irreversible fracture sites impedes full elastic recovery, manifesting as elevated residual strain at zero stress upon complete unloading. Although the residual stress only increased but remained below 7%, the material still exhibited good elasticity and low cyclic damage.

**Figure 11:**
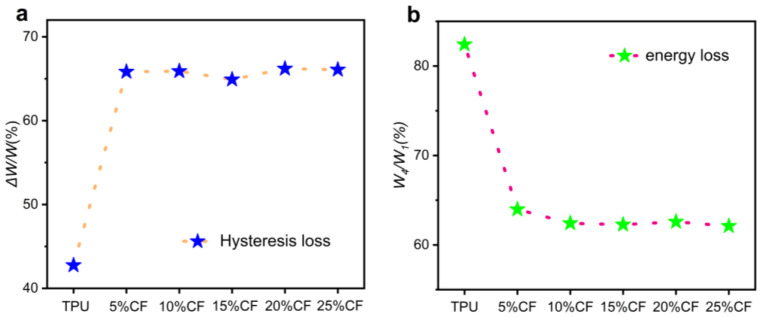
The loss of CF/TPU composites after cyclic compression: (**a**) damp loss; (**b**) energy loss.

Hysteresis loss is the ratio of the energy dissipation ΔW generated during a loading and unloading cycle to the required energy W for loading [39]. The greater the hysteresis loss, the more energy is consumed during the material loading and unloading process. The increase in hysteresis loss with fiber incorporation arises from amplified energy dissipation pathways unique to composite architectures. Carbon fibers introduce localized stress concentrations at the fiber–matrix interface, triggering reversible stick–slip motion during cyclic loading; this interfacial friction dissipates energy via Coulombic sliding, quantified by the hysteresis loop area (Figure 8). Simultaneously, fibers constrain polymer chain mobility in adjacent matrix regions, elevating the local viscoelastic relaxation rate and associated viscous losses. As shown in Figure 11a, the apparent insensitivity of hysteresis loss to fiber content stems from the TPU matrix remaining the dominant dissipation source.

To characterize the evolution of the Mullins effect in polyurethane elastomers under cyclic compression, we adopt the energy ratio metric established by Rey et al., defining the ratio of fourth-cycle loading energy W_4_ to first-cycle loading energy W_1_ [40]. This approach isolates Mullins-induced damage by negating residual strain contributions and decoupling viscoelastic dissipation (Figure 11b). The metric inherently satisfies 0 < W4/W1 ≤ 1, where lower values signify stronger Mullins softening, indicating greater irreversible microstructural damage during preconditioning. Similarly, the significant insensitivity of the first and fourth losses to fiber content in combination with hysteresis loss analysis is due to the fact that TPU matrix remains the main source of dissipation.

**Figure 12:**
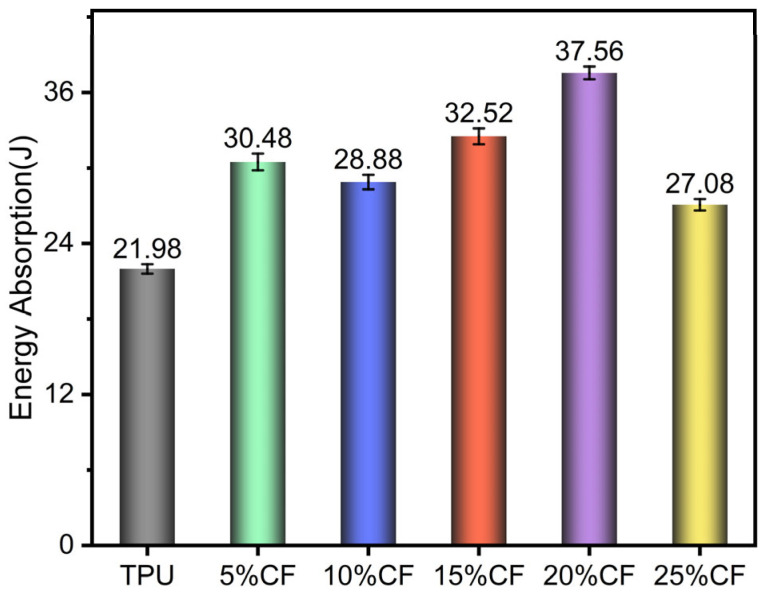
Energy absorption of compression.

Figure 12 reveals a non-monotonic energy absorption profile in CF/TPU composites under cyclic compression, peaking at 20%CF with a 70.88% enhancement over neat TPU. This maximum arises from synergistic energy storage–dissipation mechanisms. The percolated fiber network enables efficient elastic energy storage through reversible deformation, while optimized interfacial bonding facilitates stress transfer to the TPU matrix for controlled plastic dissipation via chain slippage. At these contents (5–15%CF), insufficient fiber connectivity limits reinforcement, whereas a higher content (25%CF) induces a 27.90% reduction due to agglomeration-induced defects causing premature fracture and diminished matrix contribution. Crucially, the 20% composite uniquely balances intrinsic polymer dissipation with fiber-derived elastic restitution, achieving high energy absorption.

**Figure 13:**
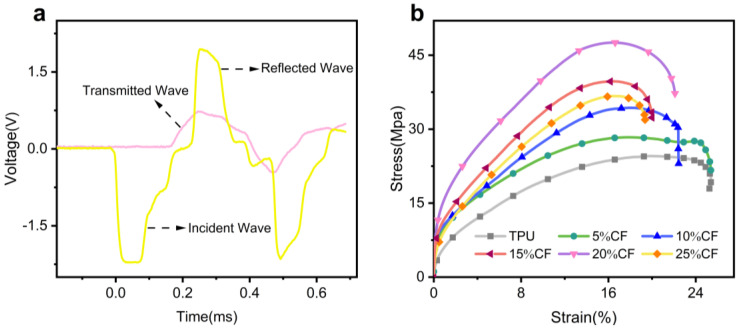
Dynamic mechanical response of CF/TPU composites under high-strain-rate compression. (**a**) The original waveform of CF/TPU dynamic impact. (**b**) Dynamic engineering stress–strain curves of CF/TPU with different fiber contents.

The effect of the alternating “rigid–elastic” structure on the mechanical properties of the composites was further analyzed by impact performance tests. To ensure valid data acquisition in split Hopkinson pressure bar (SHPB) experiments, the fundamental assumptions of one-dimensional stress wave propagation and specimen homogeneity must be satisfied. During testing, the specimen is positioned between the incident and transmission bars, with a thin layer of petroleum jelly applied at the specimen–bar interfaces to minimize frictional artifacts. A compressed gas gun propels the striker bar to impact the incident bar, generating an incident stress wave (characterized by elastic strain ε_i_) within it. As this incident wave propagates along the incident bar and reaches the specimen, a portion reflects back into the incident bar (yielding reflected strain ε_r_), while the remaining portion transmits through the specimen into the transmission bar (yielding transmitted strain ε_t_) [17,41]. Figure 13a displays the original waveform signals captured under loading at a strain rate of 10^3^ s^−1^. Crucially, as demonstrated in Figure 13a, the stress derived from the transmitted wave closely matched the sum of the stresses calculated from the incident and reflected waves throughout the loading period. This precise superposition of incident/reflected and transmitted stresses confirms the achievement of dynamic stress equilibrium within the specimen, thereby validating the homogeneity assumption essential for reliable SHPB analysis.

The relationship between the average engineering strain [ε(t)] and stress [σ(t), MPa] of the sample was calculated using the following formulas:

(2) $$\overset{\cdot }{\varepsilon }(\;t\;)=\frac{c_{0}}{l_{0}}\;(\varepsilon_{i}-\varepsilon_{r}-\varepsilon_{t})$$

(3) $$\varepsilon (\;t\;)=\frac{c_{0}}{l_{0}}\int (\varepsilon_{i}-\varepsilon_{r}-\varepsilon_{t})dt$$

(4) $$\varepsilon (\;t\;)=\frac{AE}{{2A}_{0}}(\varepsilon_{i}+\varepsilon_{r}+\varepsilon_{t})$$

In theses formulas, $\overset{\cdot }{\varepsilon }$(*t*) is strain rate, *c*_0_ is elastic wave velocity of the pressure bar (m/s), *E* is elastic modulus of the pressure bar (GPa), *A* is the cross-sectional area of the pressure bar (mm^2^), *A*_0_ is the specimen area (mm^2^), *l*_0_ is the specimen length (mm), and *t* is the loading time (ms).

**Figure 14:**
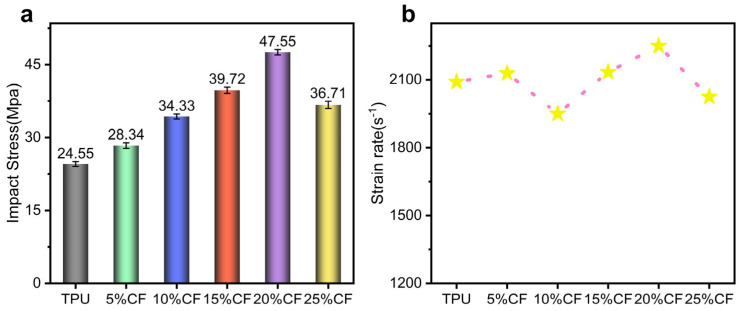
Dynamic mechanical response of CF/TPU with different fiber contents under high-strain-rate loading. (**a**) Impact strength of CF/TPU with different fiber contents. (**b**) Strain-rate of CF/TPU with different fiber contents.

To further investigate the mechanical properties of TPU under different fiber contents (0–25%), samples fabricated with optimal parameters (printing rate: 40 mm/; nozzle temperature: 220–230 °C) were subjected to high-strain-rate (Figure 14b) mechanical testing. The resulting stress–strain curve is presented in Figure 13b. As shown in Figure 13b, the CF/TPU specimen exhibits highly nonlinear behavior under dynamic compression, characterized by four distinct stages: elastic deformation, softening, strain hardening, and unloading. In the initial stage, the stress–strain ratio remains approximately constant, corresponding to elastic behavior. As strain increases, this ratio decreases relative to the initial value, indicating the onset of softening. Subsequently, stress rises sharply with increasing strain, marking the strain hardening stage. Finally, a rapid decrease in stress signifies unloading. This characteristic response arises from adiabatic compression under high-strain-rate loading [17]. A portion of the internally dissipated energy converts into heat, inducing an adiabatic temperature rise. As the temperature increases, the material undergoes densification, decreasing the distance between molecules, resulting in a thermal softening effect which increases the material’s local temperature, reducing its elastic modulus and viscosity coefficient, thereby causing the observed softening as strain further affects the molecular chains. This leads to a hardening phenomenon analogous to quasi-static hardening.

The Figure 13b and Figure 14a reveal a critical non-monotonic dependence of impact strength on CF content within the TPU matrix. At contents ≤ 20%, impact strength exhibits a positive correlation with fiber fraction, culminating in a distinct peak at 20%. This enhancement signifies effective, albeit incrementally diminishing, stress transfer and crack deflection mechanisms facilitated by the dispersed fibers below the critical threshold. However, concomitant with this rising strength is a progressive reduction in failure strain, reflecting the inherent trade-off introduced by the rigid CF phase constraining the matrix’s ductility and energy dissipation through large-scale deformation. Crucially, the precipitous drop in impact strength observed at 25% CF is mechanistically distinct and dominant; severe fiber agglomeration at this supersaturated concentration creates localized stress intensification sites. These agglomerates act as potent flaw nuclei, catastrophically initiating interfacial debonding and void coalescence under dynamic loading. This flaw-induced damage propagation rapidly overwhelms any residual reinforcing contribution, leading to premature brittle failure and the observed performance decline, consistent with the compromised mechanical properties identified in prior quasi-static testing. The 20% threshold thus represents an optimal balance between reinforcement efficacy and the onset of deleterious percolation-driven agglomeration.

**Figure 15:**
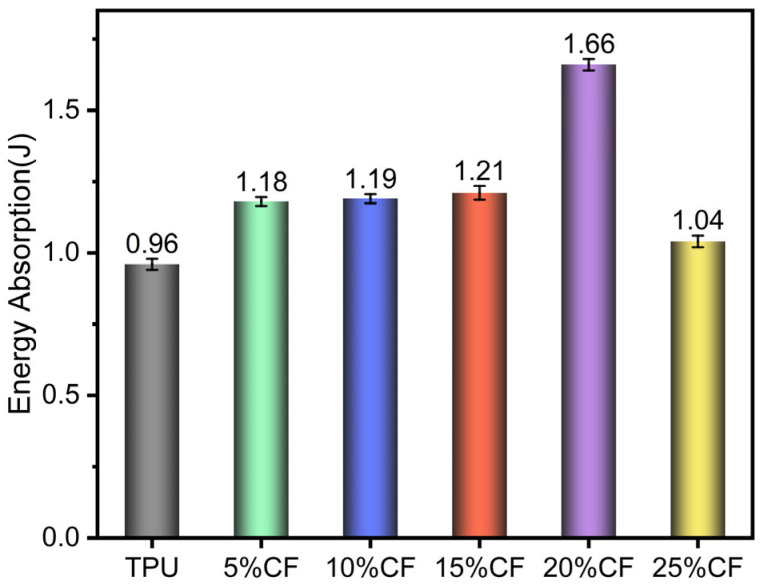
Energy absorption of impact.

The figure above displays the dynamic impact energy absorption of TPU composites reinforced with varying concentrations of CF, ranging from 0% to 25% by weight (Figure 15). At 0% CF, the TPU exhibits an energy absorption value of 0.96 J, indicating its baseline performance. As the CF content increases to 20%, there is a notable enhancement in energy absorption to 72.92%. This improvement can be attributed to the reinforcing effect of CF, which enhances the material’s mechanical properties. The addition of CF introduces a more rigid phase that effectively distributes and dissipates the impact energy, leading to a higher absorption capacity. Similarly, when fiber content reaches 25% CF loading, aggregation-induced defects promote premature fracture and compromise matrix-dominated energy dissipation mechanisms, resulting in diminished impact performance.

## 4. Conclusions

This work demonstrates the successful development and fabrication of carbon fiber-reinforced thermoplastic polyurethane (CF/TPU) composite filaments for additive manufacturing. The optimized printing parameters coupled with rational modulation of fiber mass fraction enables precise engineering of a rigid–elastic structure in composites through multiscale synergies, effectively overcoming the inherent limitations of neat TPU for structural applications.

Optimal printing parameters were achieved at 220–230 °C and 40 mm/s, balancing polymer flow for effective fiber impregnation against thermal degradation, as determined through systematic CF content (0–25%) tensile tests analysis.

Within the 0–20% carbon fiber range, the mechanical properties of CF/TPU composites exhibit a monotonic enhancement with increasing fiber mass fraction, culminating in peak performance at the 20% reinforcement threshold. However, when the fiber content exceeds 20%, fiber entanglement and agglomeration lead to a weakening trend in the mechanical properties of the material.

At 20% CF loading and printing parameters (220 °C nozzle temperature, 40 mm/s print speed), the composite compared with TPU exhibits synergistic enhancement of mechanical and energy-absorbing properties: tensile, compressive, and impact strength improve by 63.7%, 105.5%, and 93.7%, respectively, while compressive and impact energy absorption increase by 70.9% and 72.9%. The established processing–structure–property relationships reveal that carbon fibers simultaneously induce densification hardening, creating a material design paradigm for high-performance energy-absorbing applications requiring rigid–elastic functionality.

## Figures and Tables

**Table 1:** Twin screw extruder parameters of each component CF/TPU.

Sample	Main Engine Speed /r∙min^−1^	TPU Feeding Speed /r∙min^−1^	TPU Feed Rate /g∙min^−1^	Number of Fibers /n
TPU	200	4.0	34.0	0
5%CF/TPU	200	4.5	38.0	1
10%CF/TPU	200	4.2	36.0	2
15%CF/TPU	200	2.7	22.9	2
20%CF/TPU	200	2.8	23.8	3
25%CF/TPU	200	2.8	23.8	4

**Table 2:** Single-screw extruder parameters of each component CF/TPU.

Sample	Front Temperature/°C	Back Temperature/°C
TPU	213	203
5%CF-TPU	215	205
10%CF-TPU	217	207
15%CF-TPU	220	209
20%CF-TPU	223	211
25%CF-TPU	225	213

**Table 3:** Thermal parameters.

Sample	Tc/°C	Tm/°C	ΔHm/J∙g^−1^	Xc/%
TPU	90.33	250.95	4.6	3.1
5%CF/TPU	87.36	251.12	4.3	2.9
10%CF/TPU	86.81	251.06	3.9	2.6
15%CF/TPU	85.05	251.10	2.4	1.6
20%CF/TPU	83.11	251.14	2.3	1.5
25%CF/TPU	84.22	251.36	2.1	1.4

**Table 4:** Optimal printing parameters for CF/TPU with different fiber contents.

Sample	Printing Temperatures/°C	Printing Speeds/mm∙s^−1^
TPU	230	40
5%CF/TPU	230	40
10%CF/TPU	230	40
15%CF/TPU	220	40
20%CF/TPU	220	40
25%CF/TPU	220	40

## Data Availability

Data are contained within the article and supplementary materials.

## Supplementary Materials

The following supporting information can be downloaded at: https://www.mdpi.com/article/10.3390/polym17131880/s1, Figure S1: CF/TPU particles.

## References

[1] Wang P., Peng H., Huang J., Li Y., Dou Q., Suo T. (2024). Effect of flight helmet mass characteristics and neck stress postures on pilot neck impact injury. Theor. Appl. Mech. Lett..

[2] Tian J., Yang Y., Xue T., Chao G., Fan W., Liu T. (2022). Highly flexible and compressible polyimide/silica aerogels with integrated double network for thermal insulation and fre-retardancy. J. Mater. Sci. Technol..

[3] Shuguang L.I., Khan M.I., Ali F., Abdullaev S.S., Saadaoui S., Habibullah (2023). Mathematical modeling of mixed convective mhd falkner-skan squeezed sutterby multiphase flow with non-fourier heat flux theory and porosity. Appl. Math. Mech..

[4] Liu X., Ding S., Wang F., Shi Y., Wang X., Wang Z. (2022). Controlling energy dissipation during deformation by selection of the hard-segment component for thermoplastic polyure-thanes. Ind. Eng. Chem. Res..

[5] Wang A.D., Guo J.H., Shao C.K., Chen C.F. (2024). Flexible Thermoplastic Polyurethane Composites with Ultraviolet Resistance for Fused Deposition Modeling 3D Printing. 3D Print. Addict. Manuf..

[6] Oladipo B., Doner S., Lyngdoh G.A., Villada J.T., Wanchoo P., Matos H., Shukla A., Das S. (2024). Shock response of sandwich panels with additively manufactured polymer gyroid lattice cores. Mater. Today Commun..

[7] Bakır A.A., Neshani R., Özerinç S. (2021). Mechanical Properties of 3D-Printed Elastomers Produced by Fused Deposition Modeling. Fused Deposition Modeling Based 3D Printing.

[8] Płatek P., Rajkowski K., Cieplak K., Sarzyński M., Małachowski J., Woźniak R., Janiszewski J. (2020). Deformation Process of 3D Printed Structures Made from Flexible Material with Different Values of Relative Density. Polymers.

[9] Singh S., Singh R. (2020). Mechanical characterization and comparison of additive manufactured ABS, Polyflex™ and ABS/Polyflex™ blended functional prototypes. Rapid Prototyp. J..

[10] Yarwindran M., Sa’aban N.A., Ibrahim M., Periyasamy R. (2016). Thermoplastic elastomer infill pattern impact on mechanical properties 3D printed customized orthotic insole. ARPN J. Eng. Appl. Sci..

[11] Cakar S., Ehrmann A. (2021). 3D Printing with Flexible Materials-kMechanical Properties and Material Fatigue. Macromol. Symp..

[12] Bard S., Tran T., Schnl F., Rosenfeldt S., Demleitner M., Ruckdschel H. (2024). Relationship between the tensile modulus and the thermal conductivity perpendicular and in the fiber direction of PAN-based carbon fibers. Carbon. Lett..

[13] Gao X.Z., Han T.C., Tang B.L., Yi J., Cao M. (2022). Reinforced Structure Effect on Thermo-Oxidative Stability of Polymer-Matrix Composites: 2-D Plain Woven Composites and 2.5-D Angle-Interlock Woven Composites. Polymers.

[14] Zhao Y., Li Q., Wang J., Kang M., Wang X., Zhang T. (2014). Performance study of short fiber reinforced thermoplastic polyurethane elastomer composites. Fiber Compos..

[15] Georgopoulou A., Egloff L., Vanderborght B., Clemens F. (2021). A Sensorized Soft Pneumatic Actuator Fabricated with Extrusion-Based Additive Manufacturing. Actuators.

[16] Wang J., Yang B., Lin X., Gao L., Liu T., Lu Y., Wang R. (2020). Research of TPU Materials for 3D Printing Aiming at Non-Pneumatic Tires by FDM Method. Polymers.

[17] Rodríguez-Parada L., de la Rosa S., Mayuet P. (2021). Influence of 3D-Printed TPU Properties for the Design of Elastic Products. Polymers.

[18] Iacob M.C., Popescu D., Stochioiu C., Baciu F., Hadar A. (2024). Compressive behavior of thermoplastic polyurethane with an active agent foaming for 3D-printed customized comfort insoles. Polym. Testing.

[19] Shin E.J., Jung Y.S., Choi H.Y., Lee S. (2022). Synthesis and fabrication of biobased thermoplastic polyurethane filament for FDM 3D printing. J. Appl. Polym. Sci..

[20] Varsavas S.D., Kaynak C. (2018). Effects of glass fiber reinforcement and thermoplastic elastomer blending onthe mechanical performance of polylactide. Compos. Commun..

[21] Fu Y.T., Li J., Guo F.L., Li Y.Q., Fu S.Y. (2023). Micro-structure and tensile property analyses of 3D printed short carbon fiber reinforced PEEK composites. Compos. Commun..

[22] Wang G.S., Wang F.J., Wang H.Q., Fu R., Zhou J.M. (2024). Study on short fiber reinforcement mechanism for mechanical performance improvement of 3D-printed short-continuous carbon fiber reinforced thermoplastic composites. Polym. Compos..

[23] Tanabi H. (2022). Investigation of the shear properties of 3D printed short carbon fiber-reinforced thermoplastic composites. J. Thermoplast. Compos. Mater..

[24] Wang P., Zou B. (2022). Improvement of Heat Treatment Process on Mechanical Properties of FDM 3D-Printed Short- and Continuous-Fiber-Reinforced PEEK Composites. Coatings.

[25] Wang X., Tang W., Wang B., Wang L. (2012). Precise Control of Fiber Content in Fiber Reinforced thermoplastic. Aerosp. Mater. Technol..

[26] Zhai H., Li X., Yu S., Wang J., Zhou L., Xiong X. (2024). 4D printing of Nd-Fe-B composites with both shape memory and permanent magnet excitation deformation. Compos. Part. Appl. Sci. Manuf..

[27] Alzahrani M., Alhumade H., Simon L., Yetilmezsoy K., Madhuranthakam C.M.R., Elkamel A. (2023). Additive Manufacture of Recycled Poly(Ethylene Terephthalate) Using Pyromellitic Dianhydride Targeted for FDM 3D-Printing Applications. Sustainability.

[28] Frick A., Rochman A. (2004). Characterization of tpu-elastomers by thermal analysis (DSC). Polym. Test..

[29] Liu Z., Chen K., Yan D. (2003). Crystallization, morphology, and dynamic mechanical properties of poly(trimethylene terephthalate)/clay nanocomposites. Eur. Polym. J..

[30] Kanbur Y., Tayfun U. (2019). Development of multifunctional polyurethane elastomer composites containing fullerene: Mechanical, damping, thermal, and flammability behaviors. J. Elastomers Plast..

[31] Tikhani F., Gurbin A., Hubert P. (2024). Unveiling the impact of short fibre reinforcement and extrusion properties on microstructure of 3D printed polycarbonate composites. Addit. Manuf..

[32] Lei J., Shen Q., Liu T., Sun H., Yin Z. (2022). Influence of fused deposition process parameters on static and dynamic mechanical properties of thermoplastic polyurethane elastomer. Cina. Plast..

[33] Brancewicz-Steinmetz E., Brancewicz-Steinmetz E., Sawicki J., Sawicki J., Byczkowska P., Byczkowska P. (2021). The Influence of 3D Printing Parameters on Adhesion between Polylactic Acid (PLA) and Thermoplastic Polyurethane (TPU). Materials.

[34] Gallup L., Trabia M., O′Toole B., Fahmy Y. (2025). Influence of Fused Deposition Modeling Process Parameters on Constitutive Model of Hyperelastic Thermoplastic Polyurethane. Polymers.

[35] Desai S.M., Sonawane R.Y., More A.P. (2023). Thermoplastic polyurethane for three-dimensional printing applications: A review. Polym. Adv. Technol..

[36] Arifvianto B., Iman T.N., Prayoga B.T., Dharmastiti R., Suyitno S. (2021). Tensile properties of the fff-processed thermoplastic polyurethane (tpu) elastomer. Int. J. Adv. Manuf. Technol..

[37] Zheng H., Zhu S., Li Z. (2016). Continuous Deformation Monitoring by Polymermatrix Carbon Fiber Sensitive Layer. J. Wuhan Univ. Technol..

[38] León-Calero M., Vales S.C.R., Marcos-Fernandez A., Rodriguez-Hernandez J. (2021). 3D Printing of Thermoplastic Elastomers: Role of the Chemical Composition and Printing Parameters in the Production of Parts with Controlled Energy Absorption and Damping Capacity. Polymers.

[39] Ma Y., Li Z., Li X., Zhu S., Yan S. (2024). Experimental study on the cyclic mechanical behavior of polyurethane at different compression strains. J. Exp. Mech..

[40] REY T., Chagnon G., Le Cam J.B., Favier D. (2013). Influence of the temperature on the mechanical behaviour of filled and unfilled silicone rubbers. J. Polymer Testing.

[41] Bai J.Q., Qi S.B., Xie Y.C., Yuan M.Q., Li M.L. (2025). Ballistic response of an airbag with parallel ribs under spherical projectile impact. Compos. Struct..

