# Supplementary material for: Enhancing the Energy Absorption Performance of 3D-Printed CF/TPU Composite Materials by Introducing a “Rigid–Elastic” Structure Through Multi-Scale Synergies
Source: Polymers (Basel). 2025 Jul 6;17(13):1880. doi: 10.3390/polym17131880 (PMC12251667; doi:10.3390/polym17131880)
Supplement: Supplementary file 1 [file polymers-17-01880-s001.zip › polymers-3723218-supplementary.pdf]

**Preparation of CF/TPU particles**

TPU pellets were pre-dried at 75°C for 24 hours in a forced-air convection oven (DHG-9426A, Shanghai Jinghong) to achieve <0.1% moisture content. Granulation was conducted using a co-rotating twin-screw extruder (SHJ-20, Nanjing Juli Chemical Machinery) with seven temperature-controlled zones: six barrel zones (175°C, 205°C, 205°C, 205°C, 195°C, 195°C) and a dead zone (210°C). This thermal profile ensured complete polymer melting while preventing feed-throat blockage, with Zone 1 maintained at a lower temperature to avoid premature melt adhesion. Carbon fibers were introduced downstream at Zone 3 following TPU plastication to achieve homogeneous dispersion. By regulating feed rate and screw speed, TPU was uniformly blended with pristine CF at mass fractions of 0, 5, 10, 15, 20, and 25%. The extruded CF/TPU filaments were subsequently pelletized into 2–3 mm particles (Figure S1).

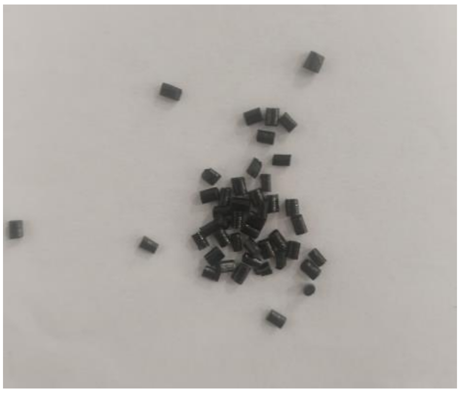

**Figure S1. Particles of CF/TPU**
